# Clinical Expression of an Inherited Unbalanced Translocation in Chromosome 6

**DOI:** 10.1155/2011/396450

**Published:** 2011-09-25

**Authors:** Bani Bandana Ganguly, Vijay Kadam, Nitin N. Kadam

**Affiliations:** ^1^MGM Center for Genetic Research & Diagnosis, MGM's New Bombay Hospital, Navi Mumbai 400703, India; ^2^Department of Medicine, Mahatma Gandhi Mission's Medical College and Hospital, Navi Mumbai 410209, India; ^3^Paediatric Department, Mahatma Gandhi Mission's Medical College and Hospital, Navi Mumbai 410209, India

## Abstract

Unbalanced chromosomal rearrangements are not common; however, they have a significant clinical expression. The parental balanced translocation produces unbalanced chromosome, which is transmitted to next generation through fertilization of gametes carrying the derivative chromosome. The carriers of balanced rearrangements mostly do not have recognizable phenotypic expression. We report a family comprising of healthy and non-consanguineous young parents and their preemie newborn severely affected with congenital anomalies and systemic disorders. Conventional Gbanding analysis of somatic chromosomes identified a balanced translocation, t(6;10)(p23;q24), in mother and an unbalanced rearrangement, der(6)t(6:10)(p23;q24)mat, in the child. The child has inherited a derivative chromosome 6 with partial deletion of 6(p23-pter) and partial trisomy 10(q24-qter), which has resulted in fusion of genes of two different chromosomes. The prominent phenotypic features of del(6p), including high forehead, flat nasal bridge, agenesis of left ear, atrial septal defect (ASD), craniosynostosis, and growth retardation, are overlapping with specific Axenfeld-Reiger-, Larsen-, and Ritscher-Sinzel/3-C syndromes, however, lacking in ocular anomalies, skeletal laxity, or cerebellar malformation. Therefore, this paper rules out the isolated effect of del(6p23) or trisomy 10(q24) on distinct previously reported syndromes and proposes the combined effect of unbalanced chromosomal alteration.

## 1. Introduction

Karyotypic-phenotypic analysis has long been a practice in understanding congenital malformation and delayed development. Balanced and unbalanced chromosome abnormalities have been noted as the underlying factor in number of syndromes and other clinical complications. Global incidence of chromosome aberration was noted as 0.3% in newborns, 5.0% in stillborns, 4.2% in 8th week of gestation, 2.4% in 12th week of gestation, 1.1% in 16th week of gestation, and 0.8% in 20th week of gestation [1]. The aberrations can occur *de novo* or through parental transmission.

Partial monosomy 6p and partial trisomy 10q are rarely seen among structural anomalies in normal population and have been described with congenital heart defect, hearing loss, ocular anomalies, craniofacial and bone deformities, growth and mental retardation, and so forth, which are overlapped with many other syndromes and chromosome aberrations. Del(6p25-pter), *de novo* or unbalanced translocation of paternal or maternal origin, has been explained as Axenfeld-Reiger syndrome [2], Larsen syndrome [3, 4], or Ritscher-Schinzel/3C syndrome [5].

In most of the cases, the parents, being the carrier of a balanced translocation involving 6p and other autosome or sex chromosome, can produce gametes with four different chromosome complements including derivative 6 (del6p) with normal copy of other chromosome, other derivative chromosome with normal 6, both rearranged chromosomes and both normal chromosomes. Therefore, inheritance of a single derivative unbalanced chromosome carries partial monosomy of one and partial trisomy of the other chromosome.

We describe a family comprising of parents and a newborn child and the effect of an unbalanced translocation with partial monosomy 6p23-pter and partial trisomy 10q24-qter rearranged on chromosome 6 at p23 of maternal origin, and with distinct craniofacial dysmorphism, ASD, pulmonary hypertension, micropenis, partial agenesis of left ear, and craniosynostosis with copper beaten skull. We discussed our case with the three syndromes reported with del6p. However, the child did not have iridodysgenesis, joint dislocation, and cerebellar malformation as reported in Axenfeld-Reiger, Larsen, and Ritscher-Schinzel syndromes, respectively. Therefore, the discussion was emphasized on genotype-phenotype association with special reference to similar cases indexed in the literature. 

## 2. Materials and Methods

### 2.1. Family and Case Report

**Figure 1:**
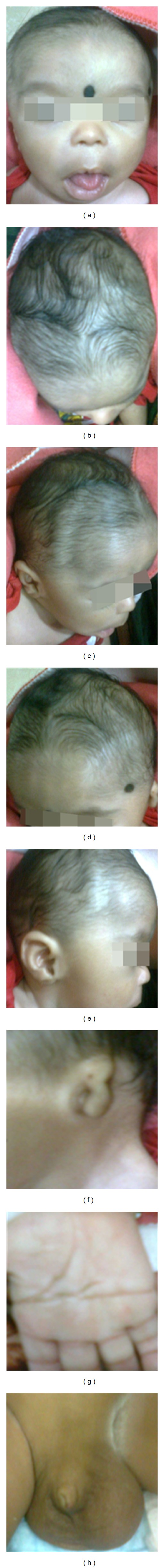
(a)–(h) Phenotype of the child at 62 days age.

A 30-day-old male child born to healthy, young and nonsanguineous parents was referred for karyotyping to rule out Down syndrome. The preemie child was born 10 days postterm to nonconsanguineous parents through uneventful vaginal delivery with cephalic presentation. The birth weight and body length were 2600 g (3rd centile) and 45 cm, respectively, with head circumference of 38 cm. However, the mother suffered continuous vomiting and abdominal pain till the end of pregnancy and experienced reduced fetal movements during ANC. The child had significant facial dysmorphic features, including flat facial profile, large fontanelle, coronal and sagital craniosynostosis, fontal prominence, flat occiput, short neck, upward slanting of eyes, hypertelorism, epicanthal fold, low set ears, left ear closed with deformed pinna, depressed nasal bridge, open mouth, high arched palate, glossoptosis micrognathia, broad philtrum, protrudent abdomen, micropenis, left-sided hydrocoele, hypotonia, increased gap between 1st and 2nd toe, atrial septal defect (ASD), Simian-like crease in right hand, and ambiguous genitalia (Figure 1).

On 30th day, the child was hospitalized due to respiratory distress and was diagnosed with right middle bronchopneumonia. Subcostal and intercoastal retraction (minimal), bilateral equal air entry, and bilateral coarse crepts (++) were noted. Metaphysis of feet was detected on X-ray. Eye examination and fundus were normal. Echo cardiogram and colour Doppler test appeared with S.D.S. congenital heart disease and a large Ostium secundum atrial septal defect (ASD) with left to right shunt. The defect was away from the mitral and tricuspid valve.

### 2.2. Chromosome Analysis

For genomic karyotyping, PHA-stimulated peripheral whole blood culture was performed in FBS (GIBCO, USA) supplemented RPMI 1640 (GIBCO, USA) medium for 72H at 37°C followed by harvesting of cells via standard colchicine-hypotonic-fixation technique. Metaphase slides were processed for GTG-banding and staining. Only complete and well-scattered metaphases were considered for karyotyping using IKAROS imaging software (MetaSystems, Germany). 

**Figure 2:**
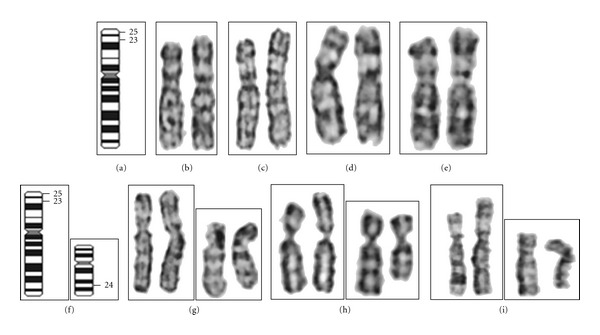
Partial karyotypes. (b)–(e) child, (g)–(i) mother (the normal chromosome is placed in left). Ideogram of chromosome 6 (L) and 10 (R).

Karyotypic analysis of 50 cells revealed 46,XY,der(6)del(6p23-pter)add(6p22) pattern in 50 cells evaluated (Figures 2(b)–2(e)).

The parents agreed for their karyotyping upon counseling. A similar protocol was followed for parents' chromosome preparation. Analysis of 25 cells from each parent revealed normal 46,XY pattern of the father, whereas the mother showed 46,XX,t(6;10)(p23;q24) pattern with a balanced translocation between 6 and 10 (Figures 2(g)–2(i)). 

Therefore, the summary of the three karyotypes was as follows: father with an apparently normal male genome, mother with a constitutive balanced translocation, t(6;10) and the child with a constitutive unbalanced translocation with der(6)t(6:10)(p23; q24)mat.

Therefore, it is apparent that the child was born through fertilization of an apparently normal paternal gamete with an abnormal maternal gamete carrying a der(6) with partial monosomy 6(p23-pter) and partial trisomy 10(q24-qter). 

## 3. Discussion

In the literature, a number of reports have been documented on balanced and unbalanced translocation in different age groups between chromosome 6 and 10 and involving many other autosomes or sex chromosomes [6–10]. Generally, the detection of unbalanced type of translocation in children with facial dysmorphism, mental retardation, and growth retardation led to retrospective detection of balanced translocations in their parents. In the present case, the child was detected with the derivative 6p which was confirmed as the product of a balanced translocation between 6p and 10q present in mother. Therefore, the derivative 6p in the present child actually designates partial monosomy 6p23 and partial trisomy 10q24 rearranged on 6p. The most of the reported cases had mild to moderate facial or craniofacial dysmorphism coupled with congenital heart defect, ocular anomalies, joint dislocation, or renal defects [11–13]. In the present family, the mother was carrying the constitutive balanced translocation and though she had some bilateral depression on the upper segment of her face, she did not have any other significant facial dysmorphism (Figure 1). Since the translocation was balanced and apparently there was no deletion or loss of genes, she did not suffer any major clinical problem since childhood. She did not have a single miscarriage so far, and she was able to achieve a pregnancy immediately after marriage with no medical intervention. However, any balanced translocation requires at least one chromosome break on each participating chromosome which might incur loss of nucleotides and obviously rearrangement of the nucleotides resulting in structural alteration of normal genes located in the two chromosomes. Therefore, an obvious disturbance at the functional level of the genes is expected in the mother with formation of new genes with new expression of new amino acid for new proteins.

The child has inherited only derivative/recombinant 6 along with normal 10 from the mother. Therefore, the gene dosage was imbalanced with partial monosomy 6(p23-pter) and partial trisomy 10(q25-qter) fused with chromosome 6 at (p22) position resulting in fusion of genes. Such unbalanced genetic make up with new chimeric fusion-gene and with few excess genes of 10(q25-qter) and loss of few genes from 6(p23) have imposed a significant phenotypic and clinical expression in the child. Nevertheless, meiotic recombination in the translocated chromosomes during maternal gametogenesis could further aggravate the expression of genetic alteration in the child. The present child had multiple craniofacial anomalies (Figure 1) and a large secundum ASD with left to right shunt and moderate to severe pulmonary hypertension and respiratory distress.

Several distinguished syndromes have been characterized with partial monosomy 6p. The craniofacial features in our case are overlapping with Axenfeld-Reiger syndrome, Larsen syndrome, and Ritscher-Schinzel or 3-C syndrome (Table 1), however, without congenital glaucoma, iris hypoplasia, hip-knee or other joint dislocations or skeletal laxity, scoliosis, cleft lip or palate, and deformities in cerebellum as reported in the three syndromes. In addition, our case had congenital heart defect with severe pulmonary hypertension, large anterior fontanelle, and craniosynostosis. The distal deletion 6p has been noted in the above-mentioned syndromes of *de novo* or parental origin. In all inherited cases, deletion was resulted from balanced translocations between 6p and many other autosomes or sex chromosomes, and del6p is speculated to be the underlying factor of the facial dysmorphism and systemic disorders. 

In addition to partial monosomy 6p, our case had partial distal trisomy 10q24-qter rearranged on 6 at p22. Since its first description by de Grouchy and Canet [14], over 50 cases have been described with distal trisomy 10q with the breakpoints ranging from 10q22.3 to 10q26.3 [15, 16]. In our case, distal trisomy10q is maternally transmitted from a balanced translocation between 6p and 10q with breakpoints at 6p23 and 10q24. Similar parental consequences have been reported in number of cases with involvement of many other autosomes or sex chromosomes [3, 16, 17]. In all such inherited cases, some other rearranged chromosome was always present mostly as monosomy. Therefore, the phenotypic manifestation is always contributed jointly by monosomy and trisomy of the two chromosomes. The distinguished characteristics recorded were mental retardation, delayed developmental milestones, hypotonia, craniofacial dysmorphism, skeletal defects of hands and feet, and defects in kidney, heart, and pulmonary systems in cases of different age groups. However, type and severity of the malformation is obviously expected to depend on the amount of loss or gain of the two chromosomes. 

The breakpoints in 6 and 10 in the present case were identified by high resolution G-banding. Due to lack of availability of FISH probes for the specific regions in two chromosomes, precise delineation of the breakpoints at subband or molecular level could not be performed in the present study. However, the breakpoints at band level are apparently correct and that obviates the deletion of *FOXC1* [5, 18–20], *FOXF1* and *FOXQ1*(5), *BMP6*, *GMDS*, *FKHL7* [21, 22] genes mapped in 6p25-pter region, whereas partial trisomy 10q24-qter confirms triplication of *PITX3* and other genes. Therefore, the complexity of phenotypic and clinical expression is solely caused by deletion or partial trisomy is not justified. Molecular mapping of the genes altered in the present case could have collected more information on genotype-phenotype association. 

Thus it is imperative to speculate that the phenotypic and systemic complication in our case could be the manifestation of the combined effect of a complex genotype with deletion of 6p, distal trisomy 10q, and chimeric gene resulted from fusion of 10q on 6p. Nevertheless, variable phenotypic and clinical expression could be due to meiotic crossing over and recombination at rearranged segments on both chromosomes and loss of nucleotides in formation of balanced translocation in mother, if any. It is apparent that the present child might develop speech, hearing and vision impairment with more systemic complications during future developmental milestones. A comprehensive followup of development could help to understand the expression of the unbalanced karyotype. Moreover, it is difficult to describe the present case as a distinct syndrome. This paper emphasizes the importance of public awareness regarding chromosomal rearrangements and importance of genetic counseling and prenatal diagnosis for prevention of recurrences and associated familial and societal stress. 

## Figures and Tables

**Table 1:** Phenotypic features recorded in different allied syndromes associated with del6p.

Phenotypic features	Our case	Reitscher-Schinzel: 3C	Axenfeld-Reiger	Larsen
Head				
High forehead	+	+		+
Hydrocephaly		+	+	
Scalp with bilateral ridges	+			
Prominent occiput		+		
Bilateral circular hair pattern	+			
Posterior fontanelle closed	+			
Anterior fontanelle		Large		
Short neck	+			+

Face				
Flat	+			+
Hypertelorism	+	+		+
Flat nasal bridge	+	+	+	+
Flat and short nose	+			
Cleft palate/lip	High arched	+ lip/palate		+ palate
Low set ears	+	+		+
Malformed ears	+	+		
Malformed ear without opening	+			
Protruding tongue	+			
Short stubby tongue	+			
Glossoptosis	+			
Down slanting palpebral fissures	+	+		
Epicanthal fold	+			
Broad philtrum	+	+		
Micrognathia	+	+		
Occular malformation	−	Coloboma	+	Iris coloboma
Glucoma	−	Inconsistent	+	+
Iris hypoplasia	−		+	−
Fundoscopy	Normal	Normal		
Bow shaped mouth	−			+
Dental abnormalities	NR		+	+

Hands				
Simian-like palmer crease	+, right hand			
Microphallus	+			
Spatulate thumbs with broad nails	−			+
Cylindrical fingers	−			+
Overriding fingers	−	+		+
Clinodactyly	+			+

Foot				
Big gap between 1st and 2nd toe	+			
Rocker bottom feet	+	+		
Club foot varus or valgus	−			+

Genitalia				
Micropenis	+			
Hydrocele	+ Left sided			
Hypospadius	−		+	+
Urogenital sinus	−			+
Pyloric stenosis	−			+
Anal atresia	−	+		+
Rectovaginal fistula	−	+		

Systemic				
Respiratory distress	+	+		
ASD/VSD	+ ASD	ASD/VSD	CHD	−
Hydronephrosis	−	+ B/L		+
Cerebellar hypoplasia	−	+	+	−
Vomiting	+			
Dilated ventricles	+			
Joint laxity	−			+
Knee/hip dislocation	−			+
Scoliosis	−			+
Psychomotor retardation	+	+	+	+
Hearing loss	? +		+/−	+
Developmental delay	+	+		+
Growth retardation	+	+	+	+
Mental retardation	+	+	+	+
Convulsions	−	−		
Immunodeficiency	+			+
Delayed speech	?+	+	+	+
